# Processing of heterogeneous MS register data within the EUReMS project

**DOI:** 10.23889/ijpds.v1i1.291

**Published:** 2017-04-19

**Authors:** Tina Meissner, Karoline Buckow, Benjamin Baum

**Affiliations:** University Medical Center Göttingen, Dep. of Medical Informatics

## Objectives

EUReMS (European Register for Multiple Sclerosis), a project including more than ten national and regional European MS registers, is aiming to enable analyses across European registers by joining existing, heterogeneous MS data in four different studies. Each participating register delivered productive data comprising information on socio-demography, disease course, medical exams or treatment. In terms of data quality, especially comparability and integrity, a data handling routine has been implemented using an open source ETL (extract transform load) tool (``Talend Open Studio'') to process the large amounts of heterogeneous raw data. That approach will be presented.

## Approach

As a first step in harmonizing datasets of different registers, a basic EUReMS data structure was defined for each of the four project studies, considering all information required to answer the research questions. Through the data handling process, the data exports are going to be converted into the prior defined study data structure to facilitate comparability and data analyses across the various registers participating in one study. In regard to quality assurance the data handling process has been validated before providing data for analyses.

## Results

The data handling process consists of five steps: Reading/Splitting, Cleaning, Mapping and Creating Study Datasets. During the first step, data is read and split into variables that are going to be used within the study datasets. The heterogeneity of the data is again noticeable in the data types of the source files, ranging from csv or Excel to Access Database. During the cleaning step, data is checked for incorrect or missing values and are, as a way of ensuring traceability, saved in specific reject files. In the mapping step, register specific variables are mapped to the defined EUReMS denotations. By that, the heterogeneous data is harmonized, disabling misinterpretation of register specific variables, often in national language or unfamiliar abbreviations. The data is merged into study datasets that are uniform in appearance for each study and are provided to the statistical department for analyses in order to gain insight on disease related questions.

## Conclusion

The implemented process enables the transparent, standardised and reproducible handling of heterogeneous data and is the groundwork for analyses across the various MS registers. Though it is a time-consuming task at the first implementation, we have been able to harmonise the heterogeneous data successfully.

